# Case of Empyema, in Which Paracentesis Was Practiced

**Published:** 1852-11

**Authors:** J. H. Beech

**Affiliations:** Coldwater, Mich.


ART. III.—*Case of Empyema, in which Paracentesis was practiced.* Reported by J. H. Beech, M. D., Coldwater, Mich.

Believing that the following case of paracentesis thoracis possesses some interest to the profession in the large amount of fluid withdrawn in proportion to the size of the patient; and that it affords much encouragement to undertake relief in forbidding cases, I am induced to offer the main facts, holding the full report of the treatment, &c., at the service of any who may take farther interest in the subject.

Wm., Jr., son of Mr. Wm. Fillison, had rubeola simplex, in February or March last. The father states that "the boy did not do well," but he could not learn from his physician that there was any complication to the measles.

Saw the patient first, May 28th, 1852. Was five years old yesterday. Much emaciated. Pulse 148, sharp and irregular. Respiration 37. Panting. Inspiration most difficult. Extremely restless. Prefers an upright position. Bowels costive. Tongue covered with thick yellow fur. Frequent hacking cough. Right shoulder higher than the left. This side of thorax much elongated; and edge of liver felt one and a half inches below the cartilages of the ribs. Whole of right thorax dull on percussion. Respiratory murmur faintly heard in upper part. Œgophony at right axillary, and infra-scapular region, when patient reclined toward left side, but not when perfectly erect. Tenderness over the infra-axillary region. Subcutaneous veins show much plainer on right than on left side. Skin hot and dry. Appetite very poor.

It seemed advisable to change the constitutional symptoms before risking an operation, and the result of medication was:

May 30, 10 o'clock, A. M. Pulse 138. Expectorates freely. Skin moist. Tongue clean at tip and edge, thin, moist, light-colored fur in middle. Appetite better. Physical signs as before. Introduced a small trocar and canula at infra-axillary region, above eighth rib, and drew with a pump fl. ℥xx. of sero-purulent fluid, when the patient complained of pain in his "side;" evinced nausea; and the pulse quickened. The canula was immediately removed, although the pleura was not quite empty.

Nine o'clock, P. M. Pulse 126, soft and full. Respiration 28. Has slept some. Tongue moist and cleaner. Sweats freely, but not excessively. Coughs less. Liver not felt below cartilage of ribs. Appetite good. Bowels have moved gently.

31st. Rested well during night. Complains of soreness of right side. No redness about the puncture. Slight excess of rotundity of right side. Crepitant râle throughout right thorax. Pulse 132.

June 4th. More cough, but still improving.

June 6th. Patient still improving in general appearance, but right thorax *nearly* as full as before. Introduced instrument near former puncture, and drew all the fluid which could be obtained by moving the canula about, and changing the position of the patient. It was greenish pus, too thick to have flowed through the canula without the pump. The last portions were tinged with blood. Patient did not complain after the trocar was introduced, and objected to lying down after the operation. Convalescence was soon fairly established, and after a few days we saw no more of him until about the middle of September, when he was hastily examined, by auscultation and percussion. No abnormal sound, or sign, was detected. He has a robust appearance.

In the above operations about six inches of flexible tube was interposed between the pump and canula, after Doct. Bowditch's plan, which effectually prevented any jerking upon the patient. The last amount of fluid drawn was fl. ℥xxii, making in all fl. ℥xlii, seven days intervening between the operations.

Coldwater, Mich., Sept. 8, 1852.

